# Prolactin is associated with bone mineral density in subjects with type 2 diabetes mellitus

**DOI:** 10.3389/fendo.2022.964808

**Published:** 2022-10-12

**Authors:** Jia Chen, Geng Liu, Quan Li, Wei Deng

**Affiliations:** ^1^ Department of Endocrinology, Beijing Jishuitan Hospital, Beijing, China; ^2^ Department of Emergency, Beijing Jishuitan Hospital, Beijing, China

**Keywords:** type 2 diabetes, prolactin, metabolism, bone mineral density, glucose

## Abstract

**Purpose:**

Prolactin (PRL) exerts actions in the bone besides lactation and reproduction. This study aimed to investigate whether PRL is related to bone mineral density (BMD) in type 2 diabetes mellitus (T2DM).

**Methods:**

A total of 642 patients with T2DM were divided into two groups with age and body mass index (BMI) matched: mildly increased PRL (HP group, n = 101) or normal PRL (NP group, n = 541). BMD was measured by dual-energy X-ray absorptiometry and compared.

**Results:**

1) BMD, T score at lumbar spine L1–4, right hip and femur neck, and Z score at the femur neck were significantly higher in the HP than in the NP group (0.96 ± 0.16 vs. 0.92 ± 0.15g/cm^2^, p = 0.019; 0.88 ± 0.15vs. 0.84 ± 0.14 g/cm^2^, p = 0.007; 0.75 ± 0.17 vs.0.70 ± 0.13 g/cm^2^, p = 0.001; -0.90 (-1.85, -0.20) vs. -1.40 (-2.20, -0.40), p = 0.018; -0.80 (-1.50, -0.30) vs. -1.10 (-1.80, -0.53), p = 0.026; -1.30 (-2.00, -0.60) vs. -1.70 (-2.20, -1.00), p = 0.001; -0.20 (-0.70, 0.30) vs. -0.40 (-0.90, 0.10), p = 0.026). In men, T and Z scores at the right hip and femur neck were significantly higher in the HP than in the NP group (-0.70 (-1.32, 0.20) vs. -0.90 (-1.50, -0.40), p = 0.038; -0.20 (-0.80, 0.20) vs. -0.50 (-0.10, 0.10), p = 0.027; -0.30 (-0.60, -0.30) vs. -0.40 (-0.90, 0.20), p = 0.038) but not in women. Bone turnover markers have no significant difference between groups (all p > 0.05). 2) BMD at the right hip and Z score at the right hip and femur neck were significantly positively associated with PRL (*r* = 0.087, p = 0.029; *r* = 0.089, p = 0.024; *r* = 0.087, p = 0.029). In men, BMD at L1–4 and the right hip; T score at L1–4, the right hip, and the femur neck; and Z score at the right hip and the femur neck were significantly positively associated with PRL (*r* = 0.122, p = 0.007; *r* = 0.105, p = 0.041; *r* = 0.123, p = 0.016; *r* = 0.110, p = 0.032; *r* = 0.115, p = 0.025; *r* = 0.121, p = 0.018; *r* = 0.138, p = 0.007) but not significant in women. 3) In men divided into two groups according to T score (T score at the right hip>-1 or T score at the right hip≤-1) or the median BMD at L1–4, the right hip or the femur neck, PRL was significantly higher in the higher BMD than in the lower BMD group (16.32 ± 6.12 vs. 14.78 ± 5.68 ng/ml, p = 0.012; 16.20 ± 6.21 vs. 14.73 ± 5.40 ng/ml, p = 0.014; 16.10 ± 6.01 vs. 14.80 ± 5.77 ng/ml, p = 0.032; 16.17 ± 6.04 vs. 14.76 ± 5.77 ng/ml, p = 0.02; 16.48 ± 6.05 vs. 14.98 ± 5.81 ng/ml, p = 0.020; 16.10 ± 5.98 vs. 14.80 ± 5.87 ng/ml, p = 0.035).

**Conclusion:**

Increased PRL was associated with better BMD in patients with T2DM, especially in men. PRL within the biologically normal range may play a protective role in the BMD of T2DM.

## Introduction

The number of people diagnosed with diabetes mellitus (DM) has rapidly increased over recent decades and DM is highly prevalent becoming a public health issue worldwide including in China (1, 2). At present, about 1 out of every 11 adults in the world have DM, and 90% of them are type 2 diabetes mellitus (T2DM) (3). Complications of T2DM mainly include diabetic nephropathy, diabetic retinopathy, and cardiovascular diseases, which are the leading causes of morbidity and mortality in T2DM patients. Additionally, T2DM is also a risk factor for fracture (3–5). Fracture risk in patients with T2DM is increased while bone mineral density (BMD) is often normal or even slightly elevated in T2DM-induced bone fragility (6). However, many studies proved that BMD was a significant predictor of fracture risk in T2DM independent of trabecular bone score and diabetes mellitus itself (7, 8). Therefore, it is important to explore the related factors that affect BMD in T2DM patients.

Prolactin (PRL) as a multifunctional hormone is involved in regulating glucose homeostasis (9). Its levels are usually higher in women than in men aged 30–50 years old (p = 0.022) (10). A study investigated the circulating PRL levels and the incident T2DM cases from 156,140 person years of follow-up (11). A total of 699 cases were documented and the results showed that a high circulating PRL level was associated with a lower T2DM risk within 9–10 years of follow-up (11). However, another cross-sectional analysis that enrolled 3,993 individuals (2,027 women) aged 20–79 years found no causal role of PRL as a risk factor for T2DM (12). Additionally, a study found that obese patients who had increased PRL within the normal range may associate with improved glucose and lipid metabolism compared to obese patients with normal PRL levels (13). Overall, the effects of PRL on T2DM are controversial and further investigation is needed.

As to the correlation between PRL and the bone, a previous study showed a lack of clinical evidence that normalization of prolactin levels in postmenopausal women improves bone mineral density or reduces the risk of fracture (14). Their association was verified in patients with stabilized schizophrenia and prolactinomas (15, 16). PRL may have direct and indirect effects on bone metabolism (17). Hyperprolactinemia caused by pituitary diseases affects bone turnover with increased bone resorption and suppressed bone formation (17). However, few studies investigated the effects of increased PRL in patients with metabolic disorders on the bone. PRL acts on target tissues through prolactin receptors (RPLR). PRLR exists in the gastrointestinal tract, kidneys, and skeletal system, which regulate calcium metabolism (18–21). Knockout of PRLR in mice leads to decreased bone formation and reduced BMD (21). Therefore, PRL may affect BMD by acting as a calcium-regulating hormone (22).

There is no study that investigated the association between normal increased PRL levels and BMD in patients with T2DM. To elucidate whether the slightly increased PRL affects BMD in T2DM patients, we conducted this cross-sectional study.

## Materials and methods

### Study protocol

This cross-sectional study enrolled patients with T2DM from the Department of Endocrinology of Beijing Jishuitan Hospital. A total of 642 T2DM patients who met the inclusion and exclusion criteria were included. T2DM was diagnosed according to the criteria from American Diabetes Association (ADA) as follows: fasting plasma glucose levels (FPG) ≥7.0 mmol/l or 2-h post-load ≥11.1mmol/l (23). Inclusion criteria were as follows: 1) Postmenopausal women with T2DM or 2) men with T2DM over 50 years old. Exclusion criteria are as follows:1) PRL over 100 ng/ml; 2) Pituitary or hypothalamic diseases; 3) taking drugs that may affect PRL levels (such as antipsychotic drugs, tricyclic antidepressants, opiates, antiemetics, protease inhibitors, and estrogens); 4) clinical or laboratory evidence of severe liver, cardiac, or renal dysfunction; 5) cancer, autoimmune diseases, acute or chronic inflammatory diseases, and other severe systemic diseases; 6) pregnancy or lactation; and 7) taking drugs that may affect bone metabolism (corticoids, thiazides diuretics, or anticonvulsants). This study was approved by the ethics committee of Beijing Jishuitan Hospital and all subjects enrolled signed the written informed consent.

### Subjects

Patients enrolled with T2DM were divided into two groups: patients with mildly increased PRL (HP group, n = 101) and patients with normal PRL (NP group, n = 541). They were age- and body mass index (BMI)-matched.

Hyperprolactinemia was defined as the following by gender: fasting PRL levels ≥20 ng/mL in men and fasting PRL levels ≥25 ng/mL in women above 2 h after waking up (24, 25). The diagnostic criteria for osteoporosis were as follows: postmenopausal women and older men with a T-score of ≤−2.5 at the lumbar spine, femur neck, or total hip by BMD testing (26).

### Anthropometric parameters

Date of age, gender, and history information including duration of diabetes were asked and recorded. Anthropometric measurements including height and body weight were tested by medical staff when patients were wearing light clothes and wearing no shoes. BMI was calculated with the following formula: BMI (kg/m^2^) = body weight (kg)/height^2^ (m^2^).

### Biochemical and glucose-lipid metabolic indexes

Blood samples were adopted when the subject was fasting for over 8 hours. Glucose and lipid metabolism were tested then. Glucose metabolic markers included fasting plasma glucose (FPG), fasting C peptide (FCP), and glycated hemoglobin (HbAlc). Lipid metabolic markers included total cholesterol (TCH), triglyceride (TG), high-density lipoprotein cholesterol (HDL-C), and low-density lipoprotein cholesterol (LDL-C).

### Bone metabolic indicators and bone density

Bone turnover markers including type I collagen, osteocalcin, calcitonin, parathyroid hormone (PTH), bone alkaline phosphatase (BALP), 25-hydroxy vitamin D (25(OH)VD), and total calcium (Ca) were measured. Bone mineral density (BMD, at the right hip, femur neck, and lumbar spine 1–4 (L1–L4)) was measured by dual-energy X-ray absorptiometry (DEXA). These values are presented in 100 score ratios or standardized difference tables and are termed Z score or T score. Z score was calculated using age-matched controls. The T scores for BMD were calculated by comparing with the BMD of healthy young people of the same sex.

### Data analysis

Statistical data were analyzed using the SPSS version 20.0 software (IBM Corp, Armonk, NY, USA). Continuous data were tested to see whether the data are normally distributed. Continuous data are presented as mean ± standard deviation (X ± SD) if normally distributed or presented as median (quartile, third Quartile) if non-normally distributed. A comparison of data between two groups was conducted using an independent-sample *t*-test if normally distributed. Nonparametric tests were applied for comparisons of data with non-normal distribution. The relationship between PRL and BMD was tested by Pearson’s or Spearman’s correlation coefficient depending on whether they were normally or non-normally distributed. Categorical data were expressed as percentage or number (n) and a chi-squared test was adopted for analysis. p Values less than 0.05 were considered significant.

## Results

### Clinical characteristics of the enrolled patients with T2DM

Of all the enrolled patients with T2DM, 261 were women and 381 were men. The average age was 61.68 ± 11.72 years old; 26.9% of them had osteoporosis (OS) while the prevalence of OS was 17.6% in men and 41.25% in women; 15.8% of them had a mild increase in PRL and the rate of hyperprolactinemia was 18.6% in men and 11.5% in women. The level of PRL was significantly higher in women than in men (17.60 ± 7.67 vs. 15.48 ± 5.92ng/ml, p < 0.001). Meanwhile, BMD at L1–4, the right hip, and the femur neck; T score at L1–4, the right hip, and the femur neck; and Z score at the right hip were all significantly higher in men than in women (0.98 ± 0.15 vs. 0.86 ± 0.14 g/cm^2^,p < 0.001; 0.89 ± 0.14 vs. 0.77 ± 0.12 g/cm^2^, p < 0.001; 0.76 ± 0.14 vs. 0.64 ± 0.11 g/cm^2^, p < 0.001; -1.10 (-1.90, -0.20) vs. -1.70 (-2.60, -0.90), p < 0.001; -0.90 (-1.50, -0.30) vs. -1.30 (-2.00, -0.70), p < 0.001; -1.30 (-1.90, -0.80) vs., p < 0.001; 0.40 (-1.00,0.20) vs. -0.20 (-0.70,0.40), p = 0.001). Bone metabolic markers including type I collagen, osteocalcin, PTH, 25(OH)VD, and total calcium between genders had no difference (all p > 0.05) while calcitonin was significantly higher in men than in women with T2DM (12.00 (6.00, 19.25) vs. 9.00 (4.00, 17.00), p < 0.001). Overall, BMD was higher in men than in women in subjects with T2DM.

### Glucose and lipid metabolism of patients with or without hyperprolactinemia

There was no statistical difference between the NP group and the HP group for age, duration of diabetes, height, body weight, and BMI (all p > 0.05). The age of women in the HP group was lower than in the NP group (57.25 ± 12.72 vs. 65.29 ± 10.81 years old, p = 0.003). The duration of diabetes, height, body weight, and BMI between the NP group and the HP group whether in women or men has no difference (all p > 0.05). FPG and HbAlc were slightly lower but there was no statistical difference between the HP group and the NP group (7.46 ± 3.02 vs. 8.06 ± 2.84 mmol/l; 8.02 ± 1.37 vs. 8.66 ± 2.00%, all p > 0.05). Whether in women or men, FPG and HbAlc were slightly lower in the HP group than in the NP group without a statistical difference (women:7.14 ± 1.16 vs. 7.57 ± 2.88 mmol/l; 8.58 ± 1.58 vs. 8.97 ± 1.95%; men: 7.63 ± 3.65 vs. 8.39 ± 2.78 mmol/l; 7.75 ± 1.24 vs. 8.45 ± 2.03%, all p > 0.05). As to lipid metabolism for all subjects, TCH, TG, and LDL-C were significantly lower in the HP group than in the NP group (TGH: 4.04 ± 0.93 vs. 4.52 ± 1.07mmol/l; TG: 1.23 (0.83, 1.75) vs. 1.62 (0.99, 2.39) mmol/l; LDL-C: 2.33 ± 0.85 vs. 2.69 ± 0.96 mmol/l, all p < 0.05). HDL-C was slightly higher in the HP group than in the NP group (1.19 ± 0.35 vs. 1.12 ± 0.35 mmol/l, p > 0.05). TG was significantly lower in the HP group than in the NP group in women and slightly lower in the HP group than in the NP group in men (0.87 (0.83,1.52) vs. 1.79 (1.22,2.71) mmol/l, p = 0.023; 1.36 (0.82, 1.80) vs. 1.39 (0.86, 2.10) mmol/l, p > 0.05). TCH and LDL-C were lower in the HP group than in the NP group in women or men (all p > 0.05). All the results are presented in **Table 1**. Better lipid metabolism was observed in diabetic patients with higher PLR than patients with normal PLR.

**Table 1:** Baseline characteristic between patients with or without increased prolactin.

Variables	All subjects		Men		Women	
	NP (n = 541)	HP (n = 101)	NP (n = 310)	HP (n = 71)	NP (n = 470)	HP (n = 30)
**Age, years old**	62.10 ± 11.40	59.45 ± 13.14	59.83 ± 11.29	60.35 ± 13.29	65.29 ± 10.81	57.25 ± 12.72
**Duration of diabetes**	10.00 (4.00, 20.00)	10.00 (3.00, 15.25)	10.00 (5.00, 18.00)	10.00 (4.00, 15.00)	13.00 (7.00,20.00)	10.00 (2.75,18.00)
**Height, m**	1.65 ± 0.08	1.67 ± 0.07	1.70 ± 0.06	1.71 ± 0.05	1.58 ± 0.05	1.59 ± 0.04
**Weight, kg**	68.14 ± 12.91	69.38 ± 13.36	72.07 ± 12.18	72.05 ± 13.37	62.59 ± 11.85	62.53 ± 10.77
**BMI, kg/m2**	24.76 ± 3.92	24.56 ± 3.85	24.79 ± 3.73	24.52 ± 3.87	24.70 ± 4.18	24.62 ± 3.88
**FPG, mmol/l**	8.06 ± 2.84	7.46 ± 3.02	8.39 ± 2.78	7.63 ± 3.65	7.57 ± 2.88	7.14 ± 1.16
**FCP, ng/ml**	1.84 (1.30, 2.69)	1.53 (1.13, 2.03)	1.61 (1.24, 2.64)	1.74 (1.28, 2.03)	2.15 (1.49,2.76)	1.32 (0.71,2.67)
**HbAlc, %**	8.66 ± 2.00	8.02 ± 1.37	8.45 ± 2.03	7.75 ± 1.24	8.97 ± 1.95	8.58 ± 1.58
**TCH, mmol/l**	4.52 ± 1.07	4.04 ± 0.93	4.54 ± 1.00	3.98 ± 1.03	4.62 ± 1.17	4.19 ± 0.73
**TG, mmol/l**	1.62 (0.99, 2.39)	1.23 (0.83, 1.75)	1.39 (0.86, 2.10)	1.36 (0.82, 1.80)	1.79 (1.22,2.71)	0.87 (0.83,1.52)*
**LDL-C, mmol/l**	2.69 ± 0.96	2.33 ± 0.85	2.63 ± 0.93	2.26 ± 0.92	2.77 ± 1.03	2.51 ± 0.73
**HDL-C, mmol/l**	1.12 ± 0.35	1.19 ± 0.35	1.15 ± 0.40	1.15 ± 0.32	1.08 ± 0.26	1.29 ± 0.46

Continuous data are presented as means ± standard deviations (SD) or medians (interquartile ranges, IQR) based on the data distribution. Categorical variables are presented as number. *Statistically significant (p < 0.05). NP, normal prolactin group; HP, increased prolactin group; BMI, body mass index; FPG, fasting plasma glucose; FCP, fasting C peptide; TCH, total cholesterol; TG, triglyceride; LDL-C, low-density lipoprotein; HDL-C, high-density lipoprotein.

### Comparison of bone turnover markers between patients with or without hyperprolactinemia

Bone turnover markers included bone formation and absorption. Whether in men or women, type I collagen, osteocalcin, PTH, BALP, calcitonin, and total Ca between the HP group and the NP group had no statistical difference (all p > 0.05). 25(OH)VD levels were slightly higher in the NP than in the HP group in all subjects, women, and men without a statistical difference (46.22 ± 18.93 vs. 42.48 ± 16.57 nmol/l; 47.04 ± 18.93vs. 41.79 ± 16.93 nmol/l; 45.90 ± 19.14vs. 2.98 ± 16.32 nmol/l; all p > 0.05). All the results were presented in **Table 2**. There was no significant difference in bone turnover markers between diabetic patients with higher PRL and normal PRL.

**Table 2:** Comparison of bone metabolism between patients with or without increased prolactin.

Variables	All subjects		Men		Women	
	NP (n = 541)	HP (n = 101)	NP (n = 310)	HP (n = 71)	NP (n = 470)	HP (n = 30)
**Type I collagen, ng/ml**	0.41 ± 0.23	0.37 ± 0.19	0.41 ± 0.24	0.40 ± 0.19	0.42 ± 0.23	0.30 ± 0.17
**Osteocalcin, ng/ml**	13.10 ± 5.37	12.00 ± 4.27	12.67 ± 5.63	12.14 ± 4.21	13.69 ± 4.95	11.59 ± 4.66
**PTH, pg/ml**	32.64 ± 19.90	29.92 ± 14.81	32.82 ± 20.68	30.48 ± 15.75	32.39 ± 18.85	28.36 ± 12.14
**BALP, µ/l**	118.73 ± 22.07	114.08 ± 19.35	117.73 ± 21.96	113.04 ± 19.53	120.09 ± 22.22	116.66 ± 19.17
**Calcitonin, pg/ml**	10.00 (5.25, 18.00)	12.50 (7.00, 19.75)	12.00 (6.00, 19.00)	14.00 (6.00, 20.00)	9.00 (4.00,17.00)	10.00 (7.00,19.50)
**25(OH)VD, mmol/l**	42.48 ± 16.57	46.22 ± 18.93	42.98 ± 16.32	45.90 ± 19.14	41.79 ± 16.93	47.04 ± 18.93
**Total calcium, mmol/l**	2.32 ± 0.10	2.30 ± 0.12	2.32 ± 0.09	2.27 ± 0.12	2.33 ± 0.11	2.36 ± 0.08

Continuous data are presented as means ± standard deviations (SD) or medians (interquartile ranges, IQR) based on the data distribution. Categorical variables are presented as number. NP, normal prolactin group; HP, increased prolactin group; PTH, parathyroid hormone; BALP, bone alkaline phosphatase; 25(OH)VD, 25-hydroxy vitamin D.

### Comparison of BMD between patients with or without hyperprolactinemia

**Figure 1:**
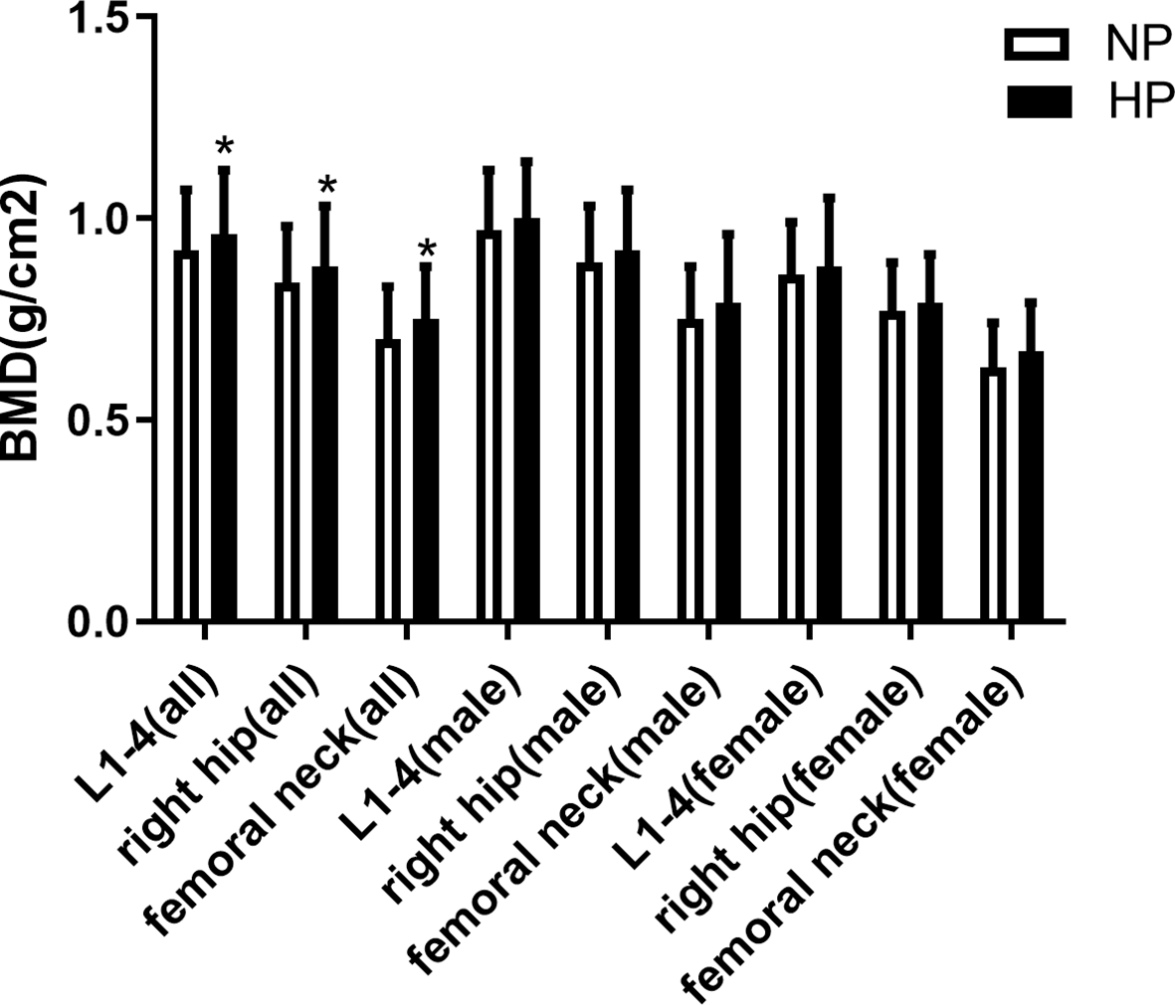
Comparison of BMD between patients with or without increased prolactin. *Statistically significant (p < 0.05).

**Figure 2:**
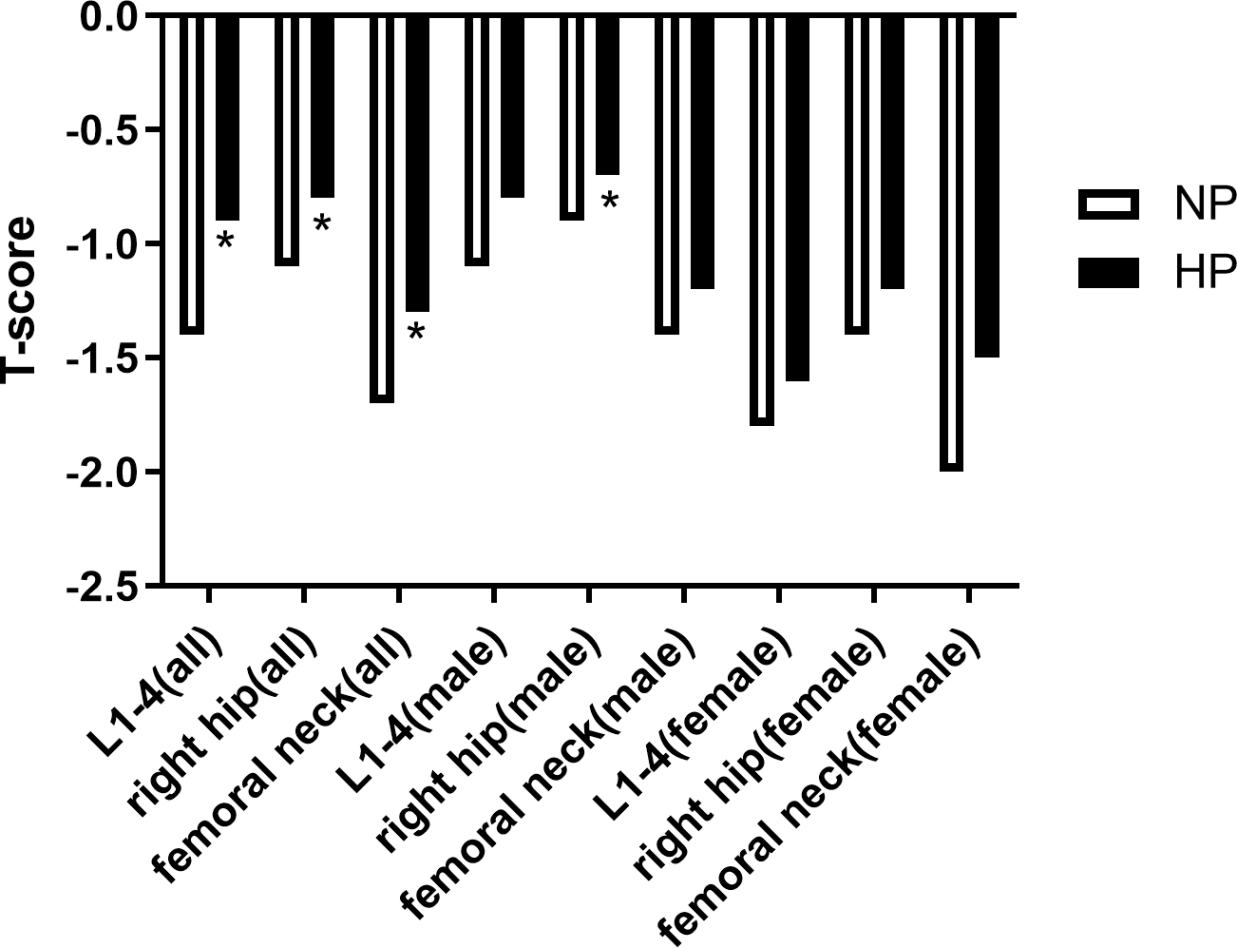
Comparison of T-score between patients with or without increased prolactin. *Statistically significant (p < 0.05).

**Figure 3:**
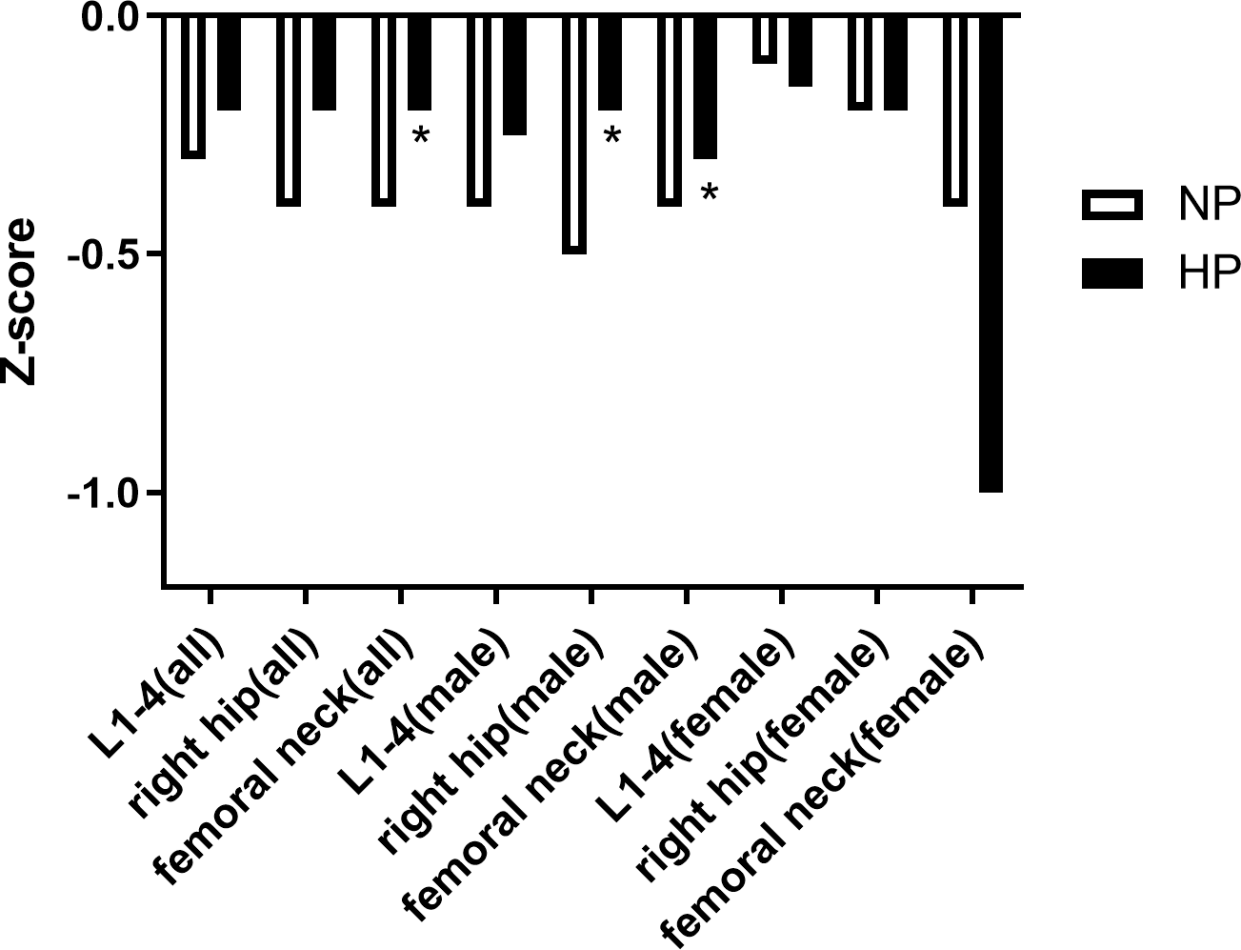
Comparison of Z-score between patients with or without increased prolactin. *Statistically significant (p < 0.05).

BMD at lumbar spine L1–4, the right hip, and the femur neck was significantly higher in the HP group than in the NP group (0.96 ± 0.16 vs. 0.92 ± 0.15 g/cm^2^, p = 0.019; 0.88 ± 0.15vs. 0.84 ± 0.14 g/cm^2^, p = 0.007; 0.75 ± 0.17 vs.0.70 ± 0.13 g/cm^2^, p = 0.001). T score at L1–4, the right hip, and the femur neck was significantly higher HP group than in the NP group (-0.90 (-1.85, -0.20) vs. -1.40 (-2.20, -0.40), p = 0.018; -0.80 (-1.50, -0.30) vs. -1.10 (-1.80, -0.53), p = 0.026; -1.30 (-2.00, -0.60) vs. -1.70 (-2.20, -1.00), p = 0.001). Z score at the femur neck was significantly higher in the HP group than in the NP group (-0.20 (-0.70, 0.30) vs. -0.40 (-0.90, 0.10), p = 0.026). Additionally, Z score at L1–4 and right hip was slightly in the HP group than in the NP group without a significant difference (-0.20 (-1.00, 0.57) vs. -0.30 (-1.07, 0.70), p > 0.05; -0.20 (-0.80, 0.30) vs. -0.40 (-0.90,0.30), p > 0.05). In men, T score at the right hip and Z score at the right hip and neck were significantly higher in p in the HP group than in the NP group (-0.70 (-1.32, 0.20) vs. -0.90 (-1.50, -0.40), p = 0.038; -0.20 (-0.80, 0.20) vs. -0.50 (-0.10, 0.10), p = 0.027; -0.30 (-0.60, -0.30) vs. -0.40 (-0.90, 0.20), p = 0.038), while BMD at L1–4, right and femur neck were slightly higher in the HP group than in the NP group without a significant difference (1.00 ± 0.14 vs. 0.97 ± 0.15 g/cm^2^,p > 0.05; 0.92 ± 0.15 vs. 0.89 ± 0.14 g/cm^2^,p > 0.05; 0.79 ± 0.17 vs. 0.75 ± 0.13 g/cm^2^,p > 0.05). T score at L1–4 and the femur neck and Z score at L1–4 were slightly higher in the HP group than in the NP group (-0.80 (-1.50, -0.20) vs. -1.10 (-1.95, -0.20), p > 0.05; -1.20 (-1.70, -0.60) vs. -1.40 (-1.92, -0.80), p > 0.05; -0.25 (-1.00, 0.52) vs. -0.40 (-1.30, 0.50), p > 0.05). The BMD in women had no difference between patients with or without increased PRL (all p > 0.05). All the results are presented in **Figures 1**, **2**, **3**. We can conclude that diabetic patients with slightly higher PRL had higher BMD than patients with normal PRL.

### Association of PRL and BMD

When analyzing the association of PRL and metabolic markers, it was shown that PRL levels were significantly negatively associated with height in all subjects (*r* = -0.104, p = 0.010) and negatively associated with TCH in men (*r* = -0.255, p = 0.044) as presented in **Table 3**. Further analysis of the association of PRL and BMD, according to the results, showed that BMD at the right hip and Z score at the right hip and the femur neck was significantly positively associated with PRL levels (*r* = 0.087, p = 0.029; *r* = 0.089, p = 0.024; *r* = 0.087, p = 0.029). In men, BMD at L1–4 and the right hip was significantly positively associated with PRL levels (*r* = 0.122, p = 0.007; *r* = 0.105, p = 0.041). T score at L1–4, the right hip, and the femur neck was significantly positively associated with PRL levels (*r* = 0.123, p = 0.016; *r* = 0.110, p = 0.032; *r* = 0.115, p = 0.025). Z score at the right hip and the femur neck was also significantly positively associated with PRL levels (*r* = 0.121, p = 0.018; *r* = 0.138, p = 0.007), but the association was not significant in women. Additionally, TCH was significantly negatively associated with PRL in men with T2DM (*r* = -0.225, p = 0.044). All the results are shown in **Table 4**. BMD and lipid metabolic marker were found to be associated with PRL in male diabetic patients.

**Table 3:** Association of prolactin and metabolism.

Variables	All subjects	Men	Women
**Age**	-0.057 (0.158)	0.016 (0.763)	-0.225 (<0.001)***
**Height**	-0.104 (0.010)*	0.036 (0.489)	-0.066 (0.309)
**Body weight**	-0.062 (0.123)	0.005 (0.927)	-0.025 (0.698)
**BMI**	-0.006 (0.103)	-0.016 (0.766)	0.003 (0.966)
**FPG**	-0.036 (0.724)	-0.009 (0.946)	-0.185 (0.259)
**FCP**	-0.011 (0.928)	-0.138 (0.372)	-0.030 (0.884)
**TCH**	-0.181 (0.071)	-0.255 (0.044)*	-0.145 (0.384)
**TG**	-0.083 (0.410)	-0.097 (0.448)	-0.176 (0.289)
**HDL-C**	-0.043 (0.676)	-0.050 (0.699)	0.174 (0.296)
**LDL-C**	-0.066 (0.512)	-0.071 (0.581)	-0.086 (0.606)
**Type I collagen**	-0.035 (0.562)	-0.002 (0.975)	-0.114 (0.231)
**Osteocalcin**	-0.014 (0.812)	0.004 (0.962)	-0.073 (0.446)
**PTH**	0.022 (0.653)	0.079 (0.218)	-0.081 (0.305)
**BALP**	-0.064 (0.190)	-0.077 (0.222)	-0.070 (0.364)
**Calcitonin**	-0.069 (0.151)	-0.016 (0.801)	-0.050 (0.519)
**25(OH)VD**	0.041 (0.397)	0.059 (0.346)	0.031 (0.685)
**Total calcium**	-0.059 (0.568)	-0.143 (0.275)	0.046 (0.782)

*Statistically significant (p < 0.05). NP, normal prolactin group; HP, increased prolactin group; BMI, body mass index; FPG, fasting plasma glucose; FCP, fasting C peptide; TCH, total cholesterol; TG, triglyceride; LDL-C, low-density lipoprotein; HDL-C, high-density lipoprotein; PTH, parathyroid hormone; BALP, bone alkaline phosphatase; 25(OH)VD, 25-hydroxy vitamin D; BMD, bone mineral density. ***Statistically significant (p < 0.001).

**Table 4:** Association of prolactin and BMD.

Variables	All subjects	Men	Women
**BMDL1–4**	0.009 (0.820)	0.122 (0.007)**	0.023 (0.717)
**T score L1–4**	0.018 (0.645)	0.123 (0.016)*	-0.007 (0.910)
**Z score L1–4**	0.035 (0.374)	0.089 (0.084)	-0.081 (0.192)
**BMD right hip**	0.087 (0.029)*	0.105 (0.041)*	0.088 (0.157)
**T score right hip**	0.042 (0.287)	0.110 (0.032)*	0.080 (0.199)
**Z score right hip**	0.089 (0.024)*	0.121 (0.018)*	0.013 (0.832)
**BMD femur neck**	0.018 (0.645)	0.084 (0.105)	0.079 (0.208)
**T score femur neck**	0.055 (0.167)	0.115 (0.025)*	0.082 (0.189)
**Z score femur neck**	0.087 (0.029)*	0.138 (0.007)**	0.016 (0.798)

*Statistically significant (p < 0.05), **statistically significant (p < 0.01). NP, normal prolactin group; HP, increased prolactin group; BMD, bone mineral density.

### PRL levels in men with different degrees of BMD

**Figure 4:**
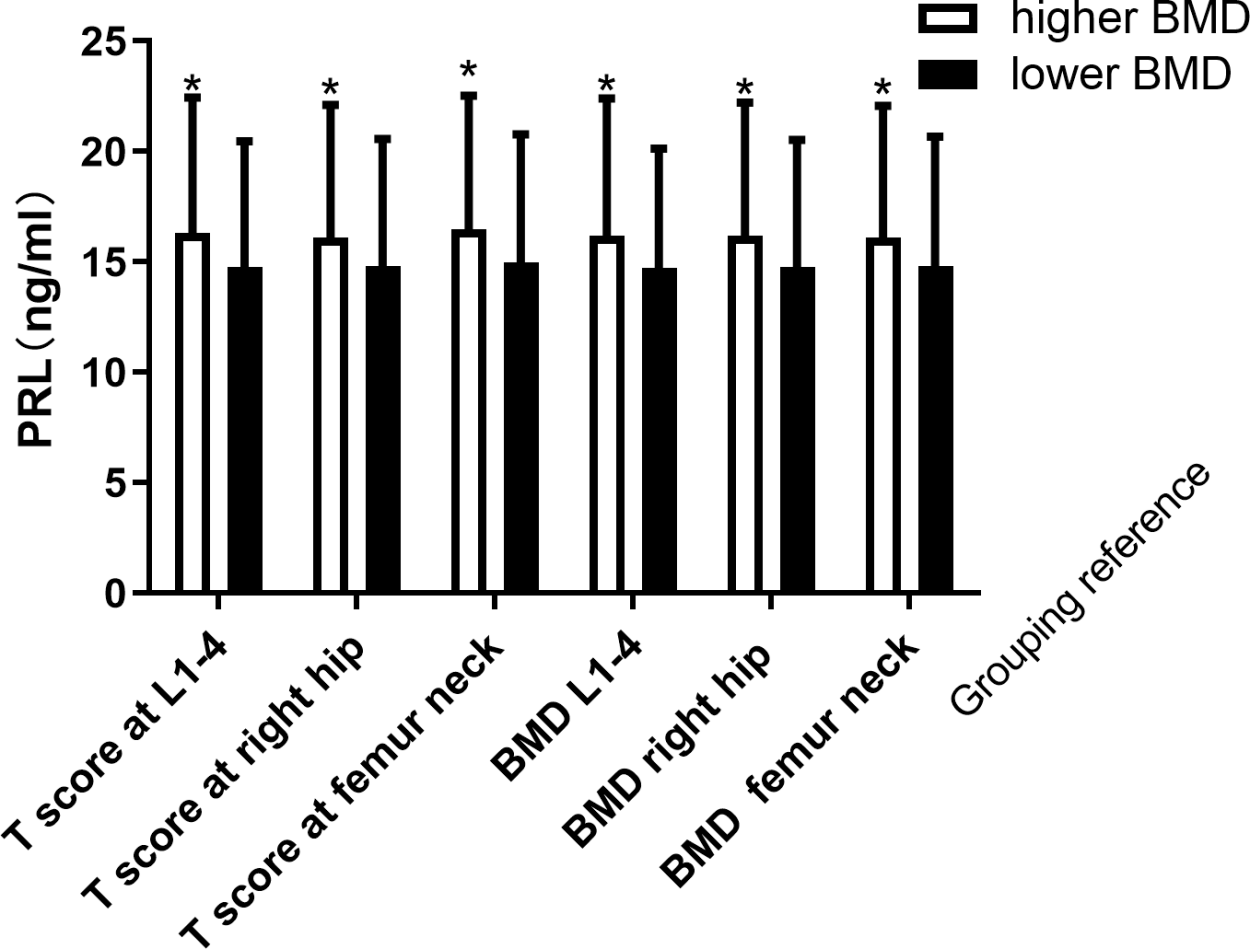
Increased PRL levels according different degree of BMD in men. *Statistically significant (p < 0.05).

As the association between PRL and BMD was more significant in men, we compared the PRL levels in men with different degrees of BMD. PRL levels were slightly higher in men with osteoporosis than in the men without osteoporosis (15.60 ± 5.93 vs. 14.26 ± 5.99 ng/ml, p = 0.109). When we divided the men into higher BMD at the right hip or lower BMD at the right hip groups according to T score (T score at the right hip >-1 or T score at the right hip ≤-1) or median BMD (BMD at the right >0.89 g/cm^2^ or BMD at the right ≤0.89 g/cm^2^). Results showed that the group with higher BMD at the right hip had higher PRL levels (16.10 ± 6.01 vs. 14.80 ± 5.77 ng/ml, p = 0.032; 16.17 ± 6.04 vs. 14.76 ± 5.77 ng/ml, p = 0.022). Also, when we divided men into higher BMD at femur neck or lower BMD at femur neck groups according to T score (T score at the right hip>-1 or T score at the right hip≤-1) or median BMD (BMD at the right>074 g/cm2 or BMD at the right ≤ 0.74 g/cm^2^). Results showed that the group with higher BMD at the femur neck had higher PRL levels (16.48 ± 6.05 vs. 14.98 ± 5.81 ng/ml, p = 0.020; 16.10 ± 5.98 vs. 14.80 ± 5.87 ng/ml, p = 0.035). Consistent with the above results, the group with higher BMD at L1–4 had higher PRL levels when men were divided into two groups according to T score (T score at the right hip>-1 or T score at the right hip≤-1) or median BMD (BMD at the right>096 g/cm^2^ or BMD at the right ≤ 0.96 g/cm^2^) (16.32 ± 6.12 vs. 14.78 ± 5.68 ng/ml, p = 0.012; 16.20 ± 6.21 vs. 14.73 ± 5.40 ng/ml, p = 0.014). All the results are presented in **Figure 4**. Overall, higher BMD was also accompanied by higher PRL in diabetic patients.

## Discussion

PRL plays an important role in maintaining glucose homeostasis (27). High serum PRL levels are associated with a low incidence of T2DM while PRL serum level is higher in patients with T2DM compared with control involved in the regulation of glucose metabolism (9). However, no study has focused on the relationship between PRL and BMD in patients with T2DM. Does increased PRL in T2DM affect BMD? With this question, we enrolled patients with T2DM to investigate the association between PRL and BMD and found the underlying relationship.

T2DM and osteoporosis are major public health concerns. Meanwhile, T2DM is a metabolic disorder including bone metabolism which impairs bone formation by increasing osteoblast apoptosis and reducing the osteoblasts and increasing the fracture risk (28). However, another study found that BMD is increased in T2DM although fracture risks are higher (29). The insulin resistance of T2DM exerts increased circulating insulin and may increase osteoblast activity and bone formation (30). As to bone turnover markers, the results of studies in diabetes are conflicting. The most consistent finding is that markers of resorption (C-terminal cross-linking telopeptide of type I collagen, N-terminal cross-linking telopeptide of type I collagen) and formation (procollagen type I N propeptide, osteocalcin) are reduced (31, 32). Histomorphometry results showed decreased bone volume, osteoid volume, thickness, and osteoblast surface and reduced bone formation indicators in T2DM (33, 34). In our study, 26.9% of the enrolled patients with T2DM had OS while the prevalence of OS was 17.6% in men and 41.25% in women. Meanwhile, PRL levels were significantly higher in women than men.

PRL is a single-chain polypeptide hormone that is synthesized and secreted by the anterior pituitary gland and targets PRLR. PRLR has been identified in the gastrointestinal tract, kidneys, and skeletal system except for mammary glands, the uterus, and ovaries (18–21). The gastrointestinal tract, kidneys, and skeletal system are involved in calcium metabolism (22). Therefore, PRL may act as a calcium-regulating hormone and affect BMD (22). PRL and PRLR play roles in bone formation and affect BMD. The knockout of PRLR leads to a decreased bone formation rate labeled by double calcein and reduced BMD measured by dual energy x-ray absorptiometry (21). In our observational study, the association between PRL and BMD had gender differences. We compared the BMD between T2DM patients with or without increased PRL. Results showed that T score at the right hip and Z score at the right hip and femur neck were significantly higher in patients with hyperprolactinemia than the patients without hyperprolactinemia in men, while the BMD in women had no significant difference between patients with or without increased PRL. Additionally, BMD at L1–4 and the right hip; T score at L1–4, the right hip, and the femur neck; and Z score at the right hip and the femur neck were significantly positively associated with PRL levels in men while the association was not significant in women. Therefore, we may infer that the association of BMD and PRL levels has gender differences. The association being more significant in men may be due to the relatively lower PRL levels when compared to women who have higher PRL levels. Upon further analysis of the PRL levels in men with different degrees of BMD, it was shown that whether in L1–4, the right hip, or the femur neck, the higher BMD of men also had significantly higher PRL levels. It was concluded that increased PRL is associated with better BMD in men. A previous animal study performed on 50 adult female rats found that hyperprolactinemia induced estrogen deficiency with a direct effect on the bone and caused bone loss in women, and estrogen and prolactin could increase serum 1,25(OH)2D3 and PTH levels (35). We infer that the women we included were mainly postmenopausal women. The decreased estrogen in them may counteract the effect of PRL on BMD. More data involving the synergistic effect of sex hormones on bone and mechanism studies are needed to explore the gender difference.

PRL also has a function on lipid metabolism. A study investigated the metabolism between obesity with or without increased PRL. The results found that TCH, LDL-C, and TG were lower in the patients with normal higher PRL than in the patients with normal PRL (all p < 0.05) (13), which is consistent with what we saw in our study. Additionally, PRL was negatively related to TCH and LDL (13). In our study which enrolled patients with men whose BMI was less than the criteria of obesity, the results also showed that PRL was significantly negatively associated with TCH in men with T2DM. The underlying mechanism needed to be further explored.

The most significant result of our study is that the increased PRL levels within normal may affect BMD in patients with T2DM, especially in men. The possible reason is that PRL may have a direct function on the bone by acting on PRLR. On the other hand, the relatively better glucose-lipid metabolism of the increased PRL within the normal range may account for the higher BMD in patients with T2DM. There also exist sex differences in these results. It is more significant in men with T2DM rather than in women. We infer that the higher PRL levels in women may affect the results. It is inferred that slightly increased PRL may have a positive effect on BMD rather than the manifested increased PRL. This study has several limitations. Firstly, the sample of the study is not larger enough and lacks follow-up results. Secondly, there is a lack of *in vitro* or animal *in vivo* experiments to further explore the underlying mechanism.

In conclusion, increased PRL within the normal range is associated with BMD in patients with T2DM, especially in men. PRL within the biologically normal range may play a protective role in the BMD of type 2 diabetes in men rather than in women.

## Data availability statement

The raw data supporting the conclusions of this article will be made available by the authors, without undue reservation.

## Ethics statement

The studies involving human participants were reviewed and approved by the ethics committee of Beijing Jishuitan Hospital. The patients/participants provided their written informed consent to participate in this study.

## Author contributions

JC designed and performed the study. GL helped to perform the experiment. QL helped to draft the manuscript. WD revised the final revision. All authors contributed to the article and approved the submitted version.

## Funding

This article was supported by the National Key Research and Development Program of China (No.2020YFC2004900).

## Conflict of interest

The authors declare that the research was conducted in the absence of any commercial or financial relationships that could be construed as a potential conflict of interest.

## Publisher’s note

All claims expressed in this article are solely those of the authors and do not necessarily represent those of their affiliated organizations, or those of the publisher, the editors and the reviewers. Any product that may be evaluated in this article, or claim that may be made by its manufacturer, is not guaranteed or endorsed by the publisher.
